# Supplementary material for: Genomic imprinting in mouse blastocysts is predominantly associated with H3K27me3
Source: Nat Commun. 2021 Jun 21;12:3804. doi: 10.1038/s41467-021-23510-4 (PMC8217501; doi:10.1038/s41467-021-23510-4)
Supplement: Supplementary file 1 — Supplementary Information [file 41467_2021_23510_MOESM1_ESM.pdf]

Supplementary Information

Genomic imprinting in mouse blastocysts is predominantly associated with H3K27me3

Laura Santini[1*], Florian Halbritter[2,3*], Fabian Titz-Teixeira[4], Toru Suzuki[5], Maki Asami[5], Xiaoyan Ma[6], Julia Ramesmayer[1], Andreas Lackner[1], Nick Warr[7], Florian Pauler[8], Simon Hippenmeyer[8], Ernest Laue[6], Matthias Farlik[3,9], Christoph Bock[3,10], Andreas Beyer[4], Anthony C. F. Perry[5#] and Martin Leeb[1#]

[1]Max Perutz Laboratories Vienna, University of Vienna, Vienna Biocenter, 1030 Vienna, Austria.

[2]St. Anna Children's Cancer Research Institute (CCRI), Vienna, Austria.

[3]CeMM Research Center for Molecular Medicine of the Austrian Academy of Sciences, Vienna, Austria.

[4]Cologne Excellence Cluster Cellular Stress Response in Aging-Associated Diseases (CECAD), University of Cologne, Cologne, Germany

[5]Laboratory of Mammalian Molecular Embryology, Department of Biology and Biochemistry, University of Bath, United Kingdom.

[6]Department of Biochemistry, University of Cambridge, United Kingdom.

[7]Mammalian Genetics Unit, MRC Harwell Institute, United Kingdom.

[8]Institute for Science and Technology Austria, Klosterneuburg, Austria

[9]Department of Dermatology, Medical University of Vienna, Vienna, Austria

[10]Institute of Artificial Intelligence and Decision Support, Center for Medical Statistics, Informatics, and Intelligent Systems, Medical University of Vienna, Vienna, Austria

*equal contribution

#Corresponding authors; equal contribution

Supplementary Figure 1. Parent-of-origin-specific gene expression analysis in blastocysts (related to Fig. 1).

Supplementary Figure 2. Parent-of-origin-specific gene expression analysis in blastocysts (related to Fig. 1).

Supplementary Figure 3. Identification of novel DMRs in uniparental embryos (related to Fig. 2).

Supplementary Figure 4. Identification of novel DMRs in uniparental embryos (related to Fig. 2).

Supplementary Figure 5. Integrated expression and methylome analysis (related to Fig. 3).

Supplementary Figure 6. Correlation of gamete-specific H3K27me3 with parental allele-specific gene expression (related to Fig. 4).

Supplementary Figure 7. Functional dependence of novel candidate genes on maternal H3K27me3 or maternal DNA methylation (related to Fig. 5).

Supplementary Figure 8. Novel imprinting clusters and novel genes in known clusters (related to Fig. 6).

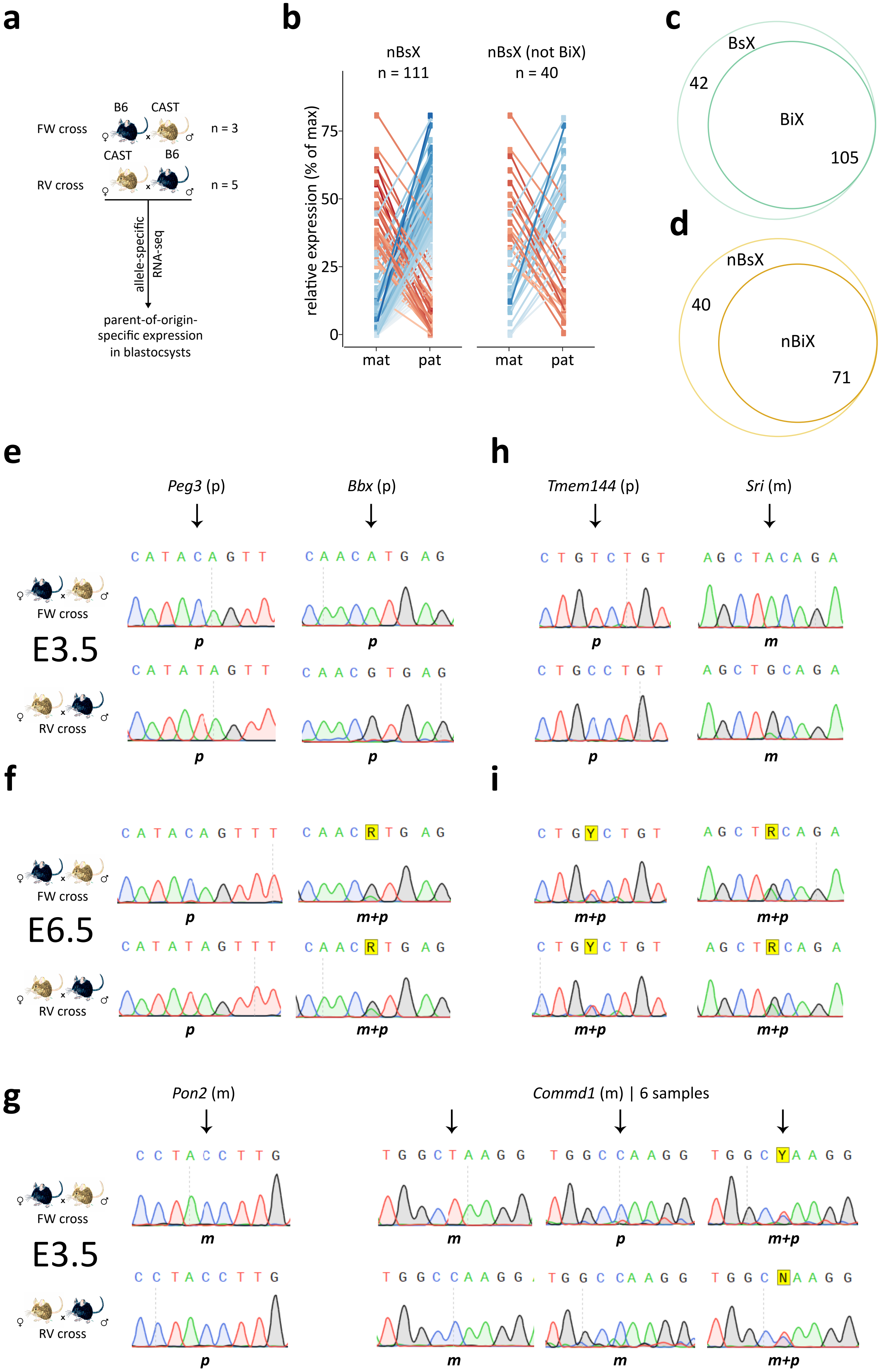

Supplementary Figure 1

Supplementary Figure 1 | Parent-of-origin-specific gene expression analysis in blastocysts (related to Fig. 1). a Schematic overview of experimental approach for the identification of parent-of-origin-specific gene expression. FW: cross between *Mus musculus domesticus* C57BL/6 (B6) female and *M. m. castaneus* (*cast*) male. RV: cross between *cast* female and B6 male. n, number of single blastocysts analysed per cross. **b** Distribution of SNP-containing RNA-seq reads between maternal and paternal alleles in nBsX genes. **c** Venn diagram showing the overlap between BsX and BiX genes. **d** Venn diagram showing the overlap between nBsX and nBiX genes. **e** Electropherogram showing RT-PCR Sanger sequencing-based validation of allele-specific expression of the confirmed published imprinted genes, *Peg3* and *Bbx* at E3.5. **f** Electropherogram showing RT-PCR Sanger sequencing-based analysis of allele-specific expression of the confirmed published imprinted genes, *Peg3* and *Bbx* at E6.5. **g** Electropherogram showing RT-PCR Sanger sequencing-based analysis of allele-specific expression of *Pon2* and *Commd1* using E3.5 embryo samples; these are published imprinted genes for which we could not confirm parent-of-origin-specific expression in blastocysts. **h** Electropherogram showing RT-PCR Sanger-sequencing-based validation of nBsX genes in E3.5 embryos. **i** Electropherogram showing RT-PCR Sanger-sequencing-based analysis of nBsX genes in E6.5 embryos. Source data are provided as Source Data files.

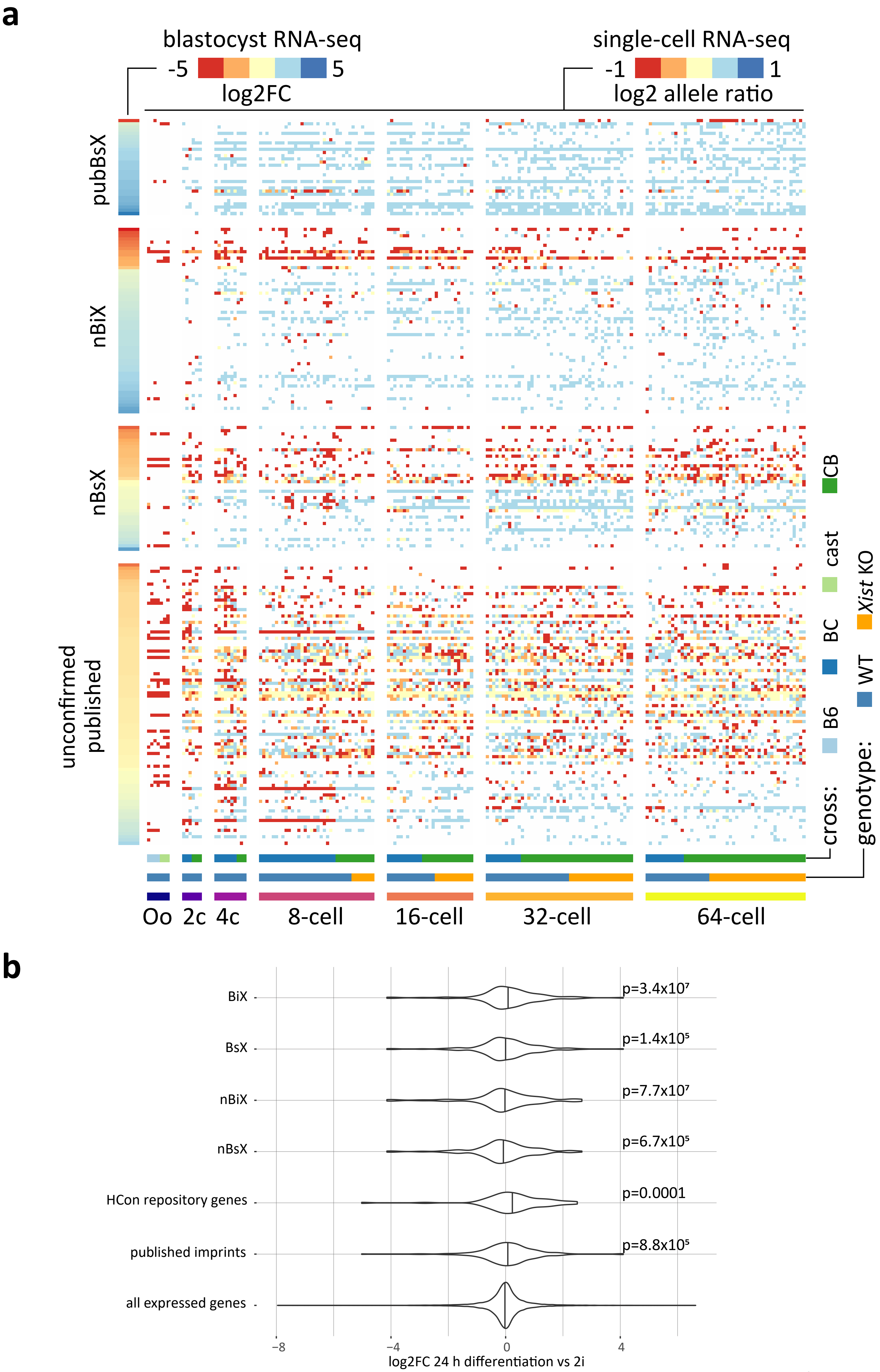

Supplementary Figure 2

Supplementary Figure 2 | Parent-of-origin-specific gene expression analysis in blastocysts (related to Fig. 1). a Heatmap visualizing single-cell RNA-seq data [1], showing parent-of-origin-specific expression of published and novel imprinted genes at the indicated stages during preimplantation development. KO refers to an *Xist* KO cell line. Colours indicate the allelic ratio of maternal and paternal reads (log2 scale).. **b** Violin plot showing absolute log2fold changes of indicated groups between ESCs (2i) and cells at an early stage of differentiation (24h after 2i withdrawal) [2]. Source data are provided as Source Data files.

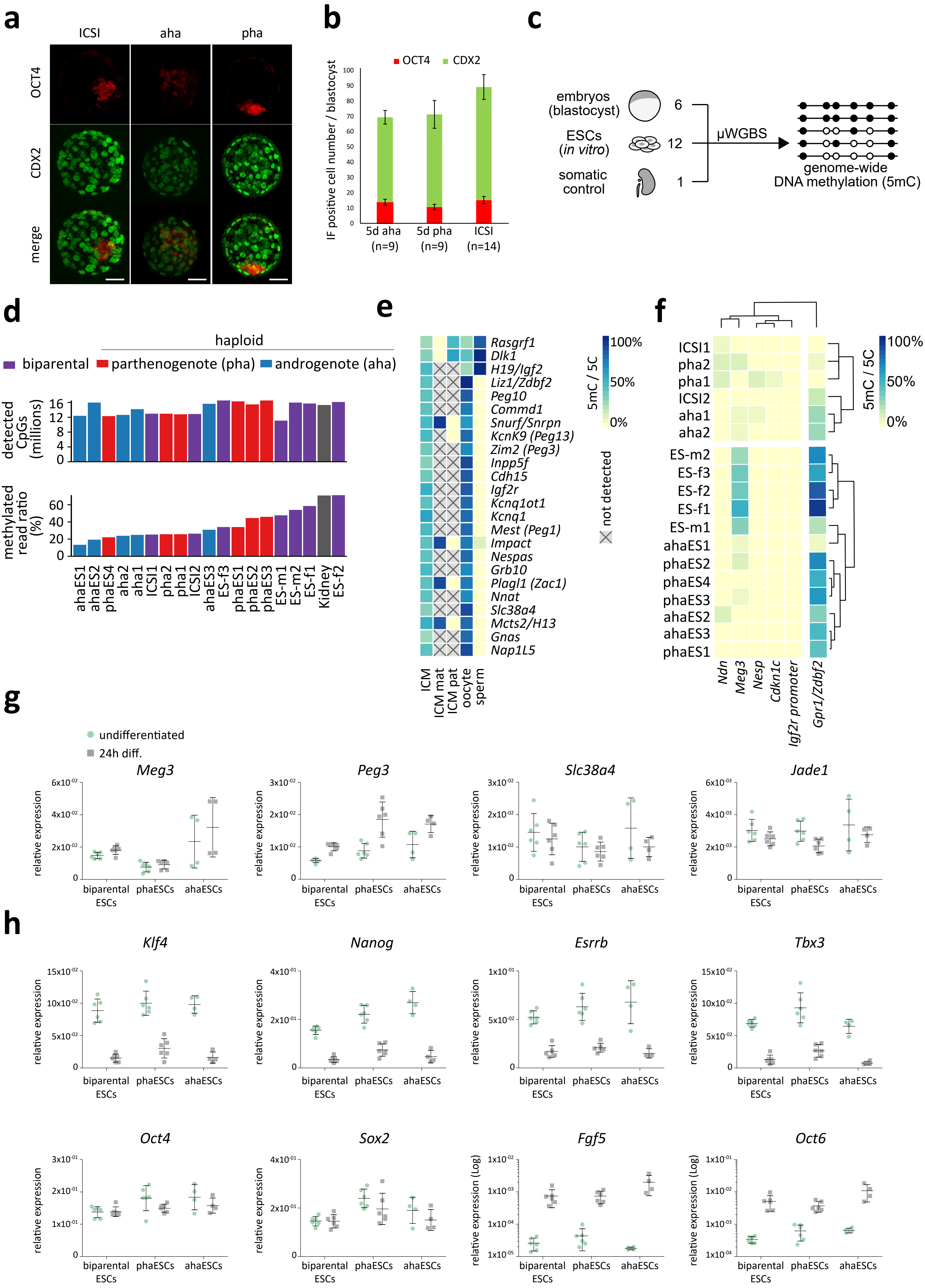

Supplementary Figure 3

Supplementary Figure 3 | Identification of novel DMRs in uniparental embryos (related to Fig. 2). a Representative immunofluorescence analysis showing OCT4 and CDX2 expression in ICSI, androgenetic haploid (andro1N) and parthenogenetic haploid (partheno1N) blastocysts. **b** Quantification of embryos of (a). Errors bars show the standard error of the means. *n*, number of independent blastocysts analysed. **c** Schematic overview of sample preparation for DNA methylation analysis. **d** Overview of number of detected CpGs (upper) and global DNA methylation levels (lower) in all samples. All regions with µWGBS signals were used as background. **e** Heatmap showing DNA methylation signals for 24 known GL-DMRs in ICM samples from previous data [3], distinguishing between maternal and paternal alleles. Grey boxes with an X indicate lack of data. Colour scale represents percentage of 5mC compared to 5C. **f** Heatmap representing DNA methylation levels from this work over known somatic DMRs. **g** RT-qPCR analysis indicating expression levels of four published imprinted genes in two androgenetic (ahaESCs), three parthenogenetic (phaESCs) and three biparental ESCs cultured in 2i (left in each pair) and for 24h after 2i withdrawal (24h diff.). Mean and standard deviation are shown for two independent experiments, with each replicate in each cell line shown as a dot. **h** RT-qPCR analysis indicating expression levels of pluripotency and early differentiation markers in two androgenetic (ahaESCs), three parthenogenetic (phaESCs), and three biparental ESCs cultured in 2i (left in each pair), and for 24h after 2i withdrawal. Mean and standard deviation are shown for two independent experiments with each replicate in each cell line shown as a dot. Source data are provided as Source Data files.

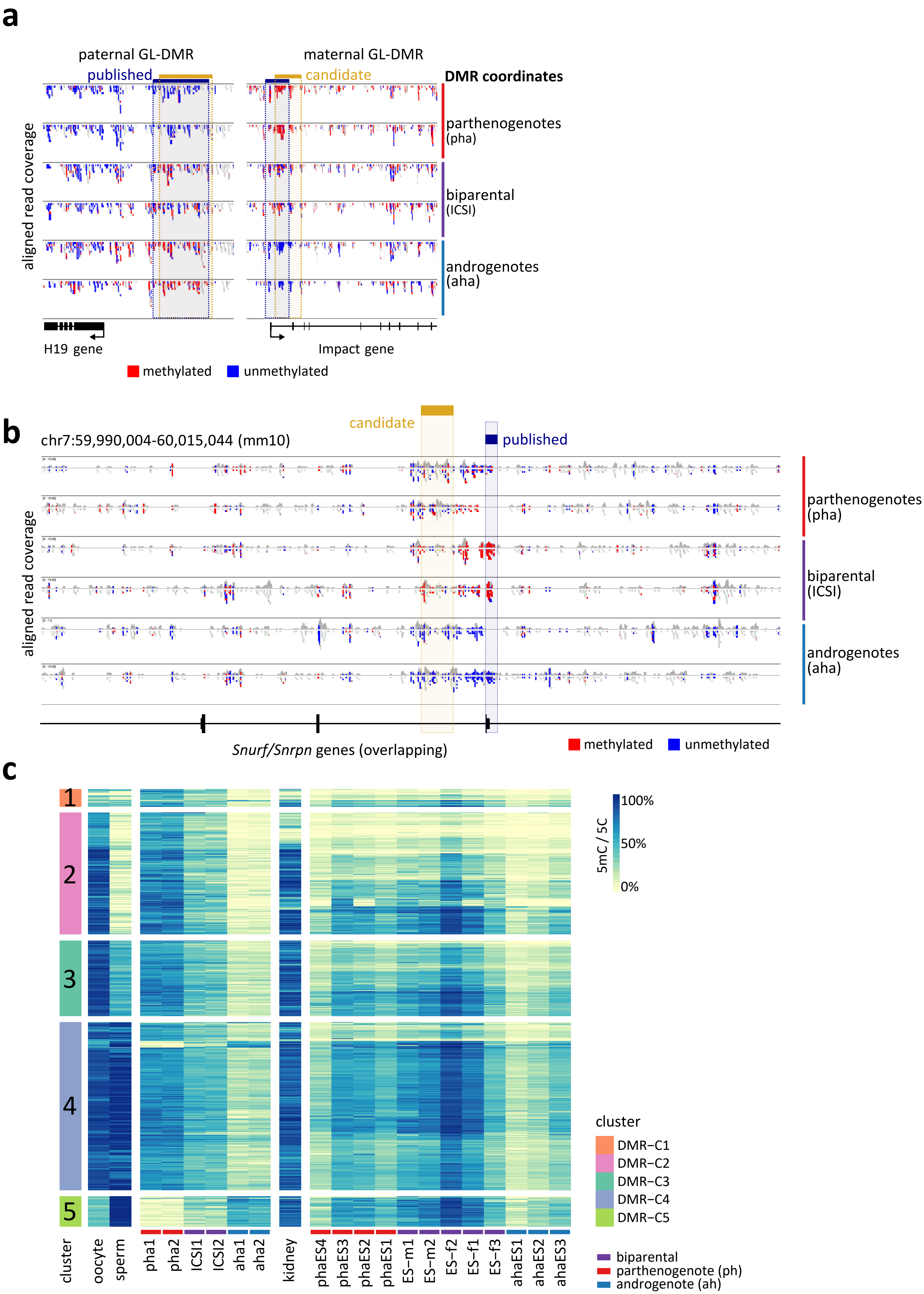

Supplementary Figure 4

Supplementary Figure 4 | Identification of novel DMRs in uniparental embryos (related to Fig. 2). a Genome browser plots showing DNA methylation signals for a paternal GL-DMR (*H19*) and a maternal GL-DMR (*Impact*). Published coordinates and coordinates determined in our analysis are indicated in blue and gold, respectively. **b** Genome browser plot showing DNA methylation signals at the *Snurf/Snrpn* locus. Published DMR coordinates and coordinates determined in our analysis are indicated in blue and gold, respectively. **c** Heatmap for all 859 DMRs identified in this work. Clustering was based on DNA methylation levels in oocyte and sperm from published datasets [3]. ESC and somatic cell DNA methylation data extend analysis from Figure 2c. Source data are provided as a Source Data files.

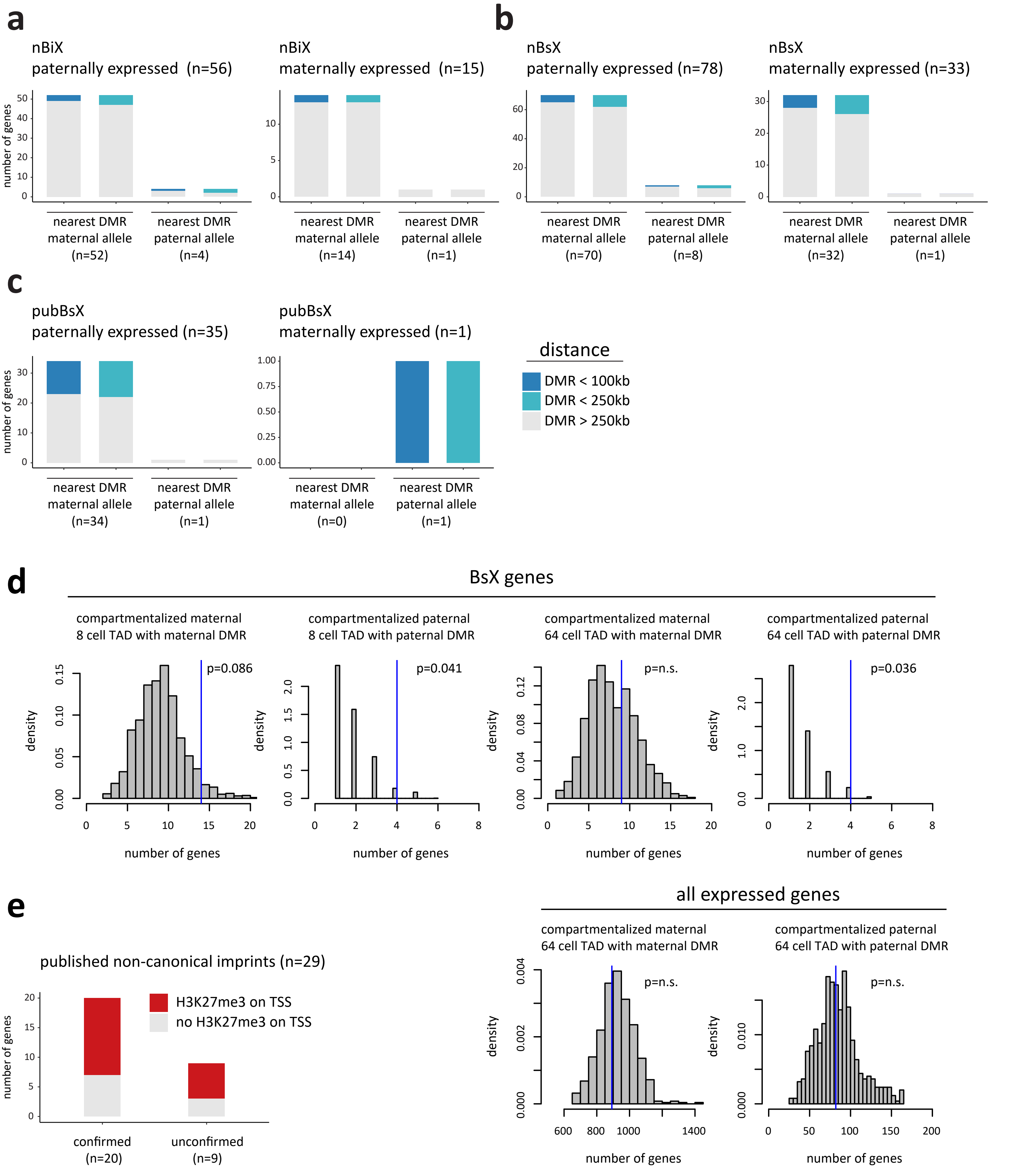
a
nBiX
paternally expressed (n=56)
nBiX
maternally expressed (n=15)
b
nBsX
paternally expressed (n=78)
nBsX
maternally expressed (n=33)
number of genes
nearest DMR maternal allele (n=52)
nearest DMR paternal allele (n=4)
nearest DMR maternal allele (n=14)
nearest DMR paternal allele (n=1)
nearest DMR maternal allele (n=70)
nearest DMR paternal allele (n=8)
nearest DMR maternal allele (n=32)
nearest DMR paternal allele (n=1)
c
pubBsX
paternally expressed (n=35)
pubBsX
maternally expressed (n=1)
nearest DMR maternal allele (n=34)
nearest DMR paternal allele (n=1)
nearest DMR maternal allele (n=0)
nearest DMR paternal allele (n=1)
distance
DMR < 100kb
DMR < 250kb
DMR > 250kb
d
BsX genes
compartmentalized maternal 8 cell TAD with maternal DMR
p=0.086
compartmentalized paternal 8 cell TAD with paternal DMR
p=0.041
compartmentalized maternal 64 cell TAD with maternal DMR
p=n.s.
compartmentalized paternal 64 cell TAD with paternal DMR
p=0.036
density
number of genes
e
published non-canonical imprints (n=29)
H3K27me3 on TSS
no H3K27me3 on TSS
number of genes
confirmed (n=20)
unconfirmed (n=9)
all expressed genes
compartmentalized maternal 64 cell TAD with maternal DMR
p=n.s.
compartmentalized paternal 64 cell TAD with paternal DMR
p=n.s.
density
number of genes

Supplementary Figure 5 | Integrated expression and methylome analysis (related to Fig. 3). **a-c** Bar charts showing associations between paternally- or maternally-expressed nBiX, nBsX and pubBsX genes with DMRs. Distances from nearest respective DMRs are colour-coded per Figure 3b. *n*, number of genes belonging to each group and displayed in the relative graph. **d** Co-occurrence of BsX genes and DMRs within the same TADs. The blue line indicates the number of BsX genes associated with at least one DMR within the same TAD at each time point. The expected distribution of the number of such genes by randomly shifting TADs coordinates (while maintaining TAD-DMR distances similar to the experimental group) is plotted as histogram in grey bars. The histogram shows the expected probability-density of observing certain number of genes co-occurring with DMR within the same TADs. Empirical *p*-values are stated. **e** Bar chart showing associations between H3K27me3-associated TSSs and published non-canonical (H3K27me3-associated) imprints [4]. Source data are provided as Source Data files.

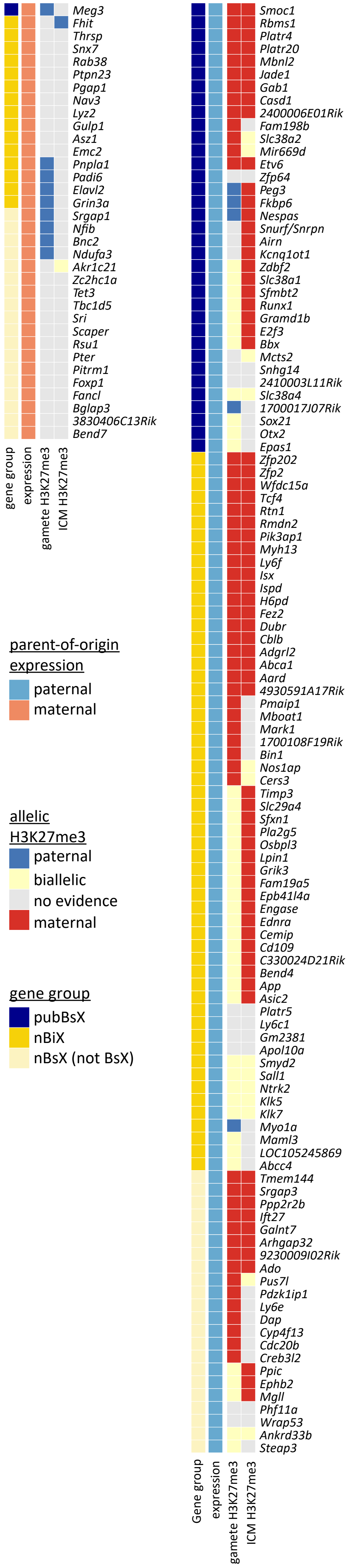

Supplementary Figure 6

Supplementary Figure 6 | Correlation of gamete-specific H3K27me3 with parental allele-specific gene expression (related to Fig. 4). Enlarged version of the heatmap of Figure 4a to show individual gene names. Source data are provided as a Source Data file.

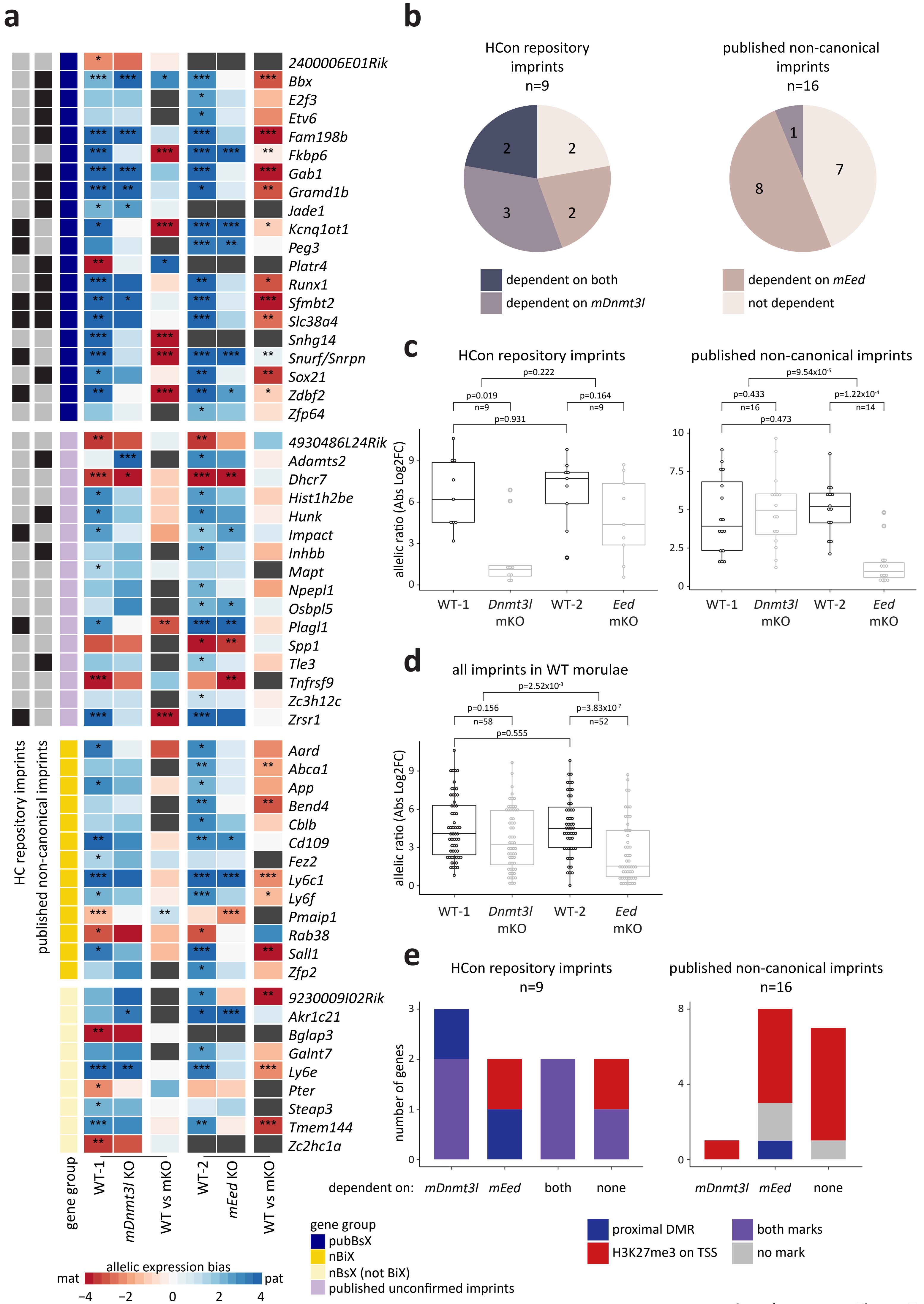

Supplementary Figure 7

Supplementary Figure 7 | Functional dependence of novel candidate genes on maternal H3K27me3 or maternal DNA methylation (related to Fig. 5). a Extended version of the heatmap of Figure 5a, including published genes for which parent-of-origin specific expression at the blastocyst stage was not confirmed (published unconfirmed imprints). Only genes with significant allelic bias (adj. $p<0.1$, DESeq2 [5]) in at least one WT morula were included in the analysis (*, adj. $p<0.1$; **, adj. $p<0.01$; ***, adj. $p<0.001$). Allelic expression bias is shown in the first two columns of each WT-mKO set (colour coded from red to blue). The third column of each WT-mKO pair indicates mKO induced changes in the allelic expression bias (colour coded from red to blue; *, adj. $p<0.05$; **, adj. $p<0.01$; ***, adj. $p<0.001$. **b** Pie charts representing the number of genes within indicated groups (HCon imprints and published non-canonical imprints) losing parent-of-origin specific expression following maternal deletion of either *Dnmt3l* (dependent on *mDnmt3l*), *Eed* (dependent on *mEed*) or both (dependent on both) in morulae. Genes not dependent on either are also indicated. **c** Box plots illustrating how allelic ratio (absolute log2FC) of high confidence repository (HCon) imprints and published non-canonical imprints are affected by maternal deletion of *Dnmt3l* (*mDnmt3l* KO) or *Eed* (*mEed* KO) at the morula stage. Only genes with significant allelic bias (adj. $p<0.1$) in at least one WT morula were included in the analysis. Paired two-tailed Wilcoxon signed rank test was performed for WT *vs* KO comparisons (WT-1 *vs mDnmt3l* KO and WT-2 *vs mEed* KO). Two-tailed Wilcoxon rank sum test were performed to compare the two WT datasets (WT-1 *vs* WT-2) and the WT *vs* KO differences between datasets. *p* values for individual comparisons are indicated in the figure. All boxplots show the 25th percentile, median and 75th percentile; whiskers indicate minimum and maximum values. **d** Box plot indicating the effect of maternal deletion of *Dnmt3l* (*mDnmt3l* KO) or *Eed* (*mEed* KO) at the morula stage on the allelic expression of all genes considered for analysis in (**a**) (BsX and published imprinted genes with significant allelic bias in at least one WT morula). Paired two-tailed Wilcoxon signed rank test was performed for WT *vs* KO comparisons (WT-1 *vs mDnmt3l* KO and WT-2 *vs mEed* KO). Two-tailed Wilcoxon rank sum tests were performed to compare the two WT datasets (WT-1 *vs* WT-2) and the WT *vs* KO differences between datasets. *p* values for individual comparisons are indicated in the Figure. All boxplots show the 25th percentile, median and 75th percentile; whiskers indicate minimum and maximum values. **e** Bar charts showing the associations between functional responses to loss of either *mDnmt3l* or *mEed* (as defined in **(b)**) with physical proximity to DMRs (within 250 kb or in the same TAD) or the presence of TSS-associated H3K27me3 (±5kb) for HCon repository imprints and published non-canonical imprints. Source data are provided as Source Data files.

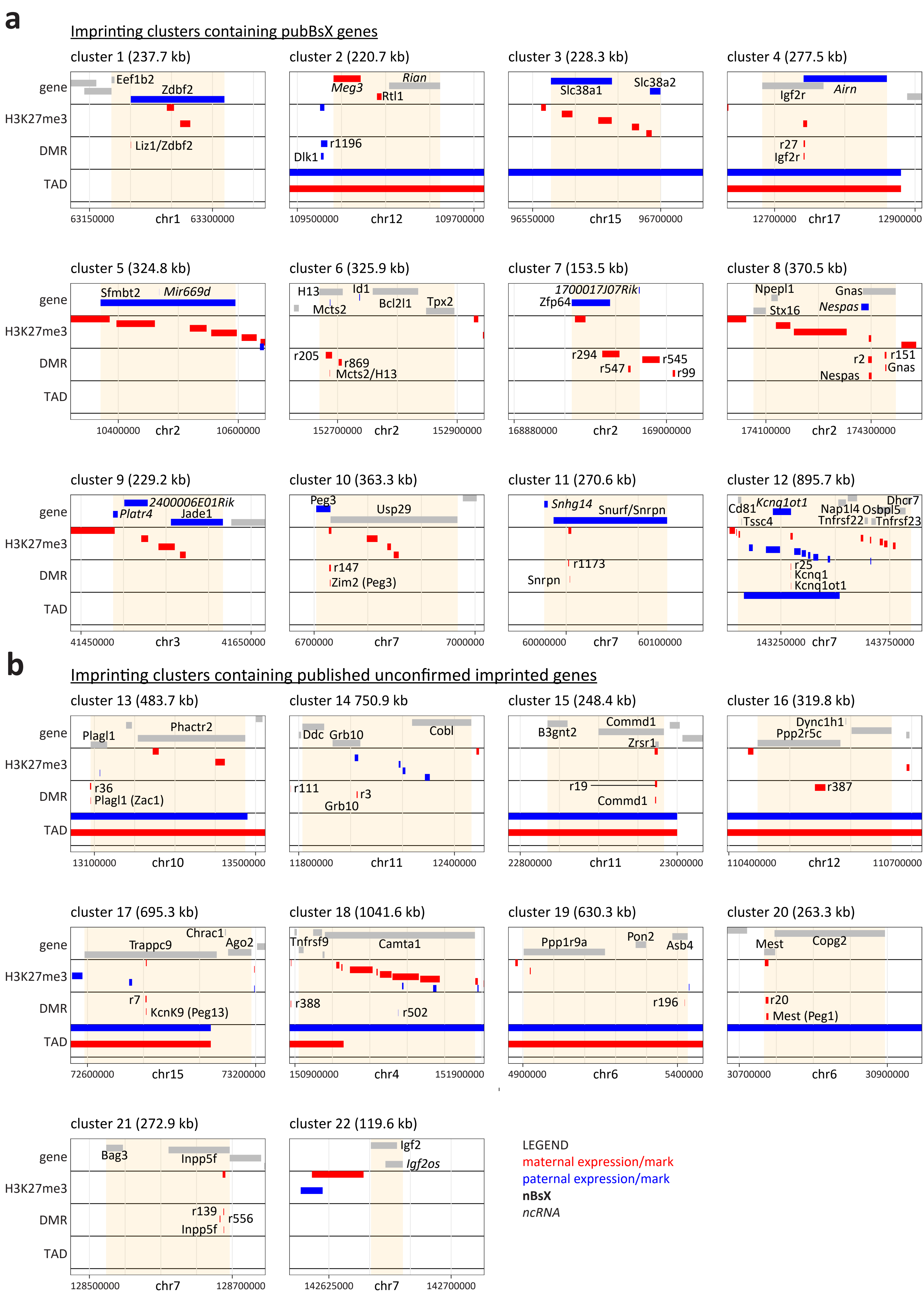

Supplementary Figure 8

Supplementary Figure 8 | Novel imprinting clusters and novel genes in known clusters (related to Fig. 6). a and **b** Close-up views as per Figure 6a and 6b, for **(a)** gene clusters containing published imprinted genes and at least one pubBsX gene (1-12) and **(b)** gene clusters containing published imprinted genes with no evidence of parent-of-origin specific expression in blastocysts (13-22). Source data are provided as a Source Data file. Red indicates maternal, and blue paternal allelic expression (genes quadrant, uppermost; based on our data), maternal/paternal H3K27me3 (H3K27me3 quadrant; based on [6]), maternal/paternal DMR (DMR quadrant; based on our data), maternal/paternal TAD (TAD quadrant; based on [7]). Grey colour for specified genes indicates published imprinted genes for which parent-of-origin specific expression was not confirmed; grey genes without gene names represent neighbouring genes not included in the cluster analysis. ncRNAs are indicated in italics. nBsX genes are indicated in bold. Source data are provided as Source Data files.

Supplementary References

1. Borensztein, M. et al. Xist-dependent imprinted X inactivation and the early developmental consequences of its failure. *Nat Struct Mol Biol* **24**, 226-233 (2017).

2. Lackner, A. et al. Cooperative genetic networks drive a mammalian cell state transition. *bioRxiv*, 2020.03.23.000109 (2020).

3. Wang, L. et al. Programming and Inheritance of Parental DNA Methylomes in Mammals. *Cell* **157**, 979-991 (2014).

4. Inoue, A., Jiang, L., Lu, F., Suzuki, T. & Zhang, Y. Maternal H3K27me3 controls DNA methylation-independent imprinting. *Nature* **547**, 419 (2017).

5. Love, M.I., Huber, W. & Anders, S. Moderated estimation of fold change and dispersion for RNA-seq data with DESeq2. *Genome Biol* **15**, 550 (2014).

6. Zheng, H. et al. Resetting Epigenetic Memory by Reprogramming of Histone Modifications in Mammals. *Molecular Cell* **63**, 1066-1079 (2016).

7. Collombet, S. et al. Parental-to-embryo switch of chromosome organization in early embryogenesis. *Nature* **580**, 142-146 (2020).
