# Supplementary material for: Genomic imprinting in mouse blastocysts is predominantly associated with H3K27me3
Source: Nat Commun. 2021 Jun 21;12:3804. doi: 10.1038/s41467-021-23510-4 (PMC8217501; doi:10.1038/s41467-021-23510-4)
Supplement: Supplementary file 3 — Description of Additional Supplementary Files [file 41467_2021_23510_MOESM3_ESM.pdf]

Description of Additional Supplementary Files

Supplementary Data 1: Catalogue of published imprinted genes

Supplementary Data 2: RNA-Seq dataset

Supplementary Data 3: Validation of imprinted genes

Supplementary Data 4: Embryo development after semi-cloning

Supplementary Data 5: μWGBS dataset

Supplementary Data 6: Primers
