# Supplementary material for: Genomic imprinting in mouse blastocysts is predominantly associated with H3K27me3
Source: Nat Commun. 2021 Jun 21;12:3804. doi: 10.1038/s41467-021-23510-4 (PMC8217501; doi:10.1038/s41467-021-23510-4)
Supplement: Supplementary file 10 — Reporting Summary [file 41467_2021_23510_MOESM10_ESM.pdf]

nature research

Corresponding author(s): Leeb, M. and Perry, A.C. F.

Last updated by author(s): March 10th, 2021

Reporting Summary

Nature Research wishes to improve the reproducibility of the work that we publish. This form provides structure for consistency and transparency in reporting. For further information on Nature Research policies, see our Editorial Policies and the Editorial Policy Checklist.

Statistics

For all statistical analyses, confirm that the following items are present in the figure legend, table legend, main text, or Methods section.

n/a	Confirmed	
[ ]	[x]	The exact sample size (*n*) for each experimental group/condition, given as a discrete number and unit of measurement
[ ]	[x]	A statement on whether measurements were taken from distinct samples or whether the same sample was measured repeatedly
[ ]	[x]	The statistical test(s) used AND whether they are one- or two-sided<br>*Only common tests should be described solely by name; describe more complex techniques in the Methods section.*
[x]	[ ]	A description of all covariates tested
[ ]	[x]	A description of any assumptions or corrections, such as tests of normality and adjustment for multiple comparisons
[ ]	[x]	A full description of the statistical parameters including central tendency (e.g. means) or other basic estimates (e.g. regression coefficient) AND variation (e.g. standard deviation) or associated estimates of uncertainty (e.g. confidence intervals)
[x]	[ ]	For null hypothesis testing, the test statistic (e.g. *F*, *t*, *r*) with confidence intervals, effect sizes, degrees of freedom and *P* value noted<br>*Give P values as exact values whenever suitable.*
[x]	[ ]	For Bayesian analysis, information on the choice of priors and Markov chain Monte Carlo settings
[x]	[ ]	For hierarchical and complex designs, identification of the appropriate level for tests and full reporting of outcomes
[ ]	[x]	Estimates of effect sizes (e.g. Cohen's *d*, Pearson's *r*), indicating how they were calculated

Our web collection on statistics for biologists contains articles on many of the points above.

Software and code

Policy information about availability of computer code

Data collection	RNA-seq/WGBS: Only standard manufacturer-provided software were used for primary sequence data collection.<br>Sanger sequencing: PCR-amplification of SNPs-containing region(s) of uniparentally expressed genes were submitted to Eurofins Genomics for Sanger sequencing analysis.
Data analysis	RNA-seq: Sequence data were mapped using STARaligner (v2.6.0.c), processed using the Allelome.Pro (v1.0) and differential analysis were performed with DESeq2.<br>WGBS: Sequence data were processed using Trimmomatic (v0.32), Bismark (v0.12.2), and Bowtie2 (v2.2.4).<br>Further analysis were performed using custom code in R (v 3.6.3) using appropriate Bioconductor packages. Notably, we used "dmrseq" for differential methylation analysis, the Enrichr web API functional enrichment analysis (date: February 2020), and "LOLA" for genomic locus overlap analysis (v1.16.0). We search DNA sequences underlying DMRs for motifs in the HOCOMOCO database (v11) using FIMO (v4.10.2). For the reanalysis of public data, we used liftOver to convert genomic coordinates to mm10, where appropriate.<br>Tissue enrichment analysis: The analysis was performed with the R package TissueEnrich (v1.10.0).<br>Sanger sequencing: Sequence data were analysed using SnapGene (v 5.0.8).<br>qPCRs: quantitative PCR analyses were performed on a BioRad CFX384 Touch qPCR machine; analyses of obtained results were performed with Microsoft Excel (Office 365) and resulting graphs were generated with GraphPad Prism (v5). ImageJ vl.50i, Fiji ver 2.0.0-rc-69/1.52p was used for image analysis.

For manuscripts utilizing custom algorithms or software that are central to the research but not yet described in published literature, software must be made available to editors and reviewers. We strongly encourage code deposition in a community repository (e.g. GitHub). See the Nature Research guidelines for submitting code & software for further information.

Data

Policy information about availability of data

All manuscripts must include a data availability statement. This statement should provide the following information, where applicable:

- Accession codes, unique identifiers, or web links for publicly available datasets
- A list of figures that have associated raw data
- A description of any restrictions on data availability

RNA-seq and WGBS data generated in this study are freely available from to the Gene Expression Omnibus (GEO, accession number: GSE152106).
Data from the following accession numbers were used in this study:
GSE56697 (MethylC-Seq data from oocytes, sperm, and ICM, https://www.ncbi.nlm.nih.gov/geo/query/acc.cgi?acc=GSE56697),
GSE76687 (allele-specific H3K27me3 peak coordinates in ICM, https://www.ncbi.nlm.nih.gov/geo/query/acc.cgi?acc=GSE76687),
GSE80810 (single-cell gene expression data from oocytes and preimplantation embryos, https://www.ncbi.nlm.nih.gov/geo/query/acc.cgi?acc=GSE80810),
GSE130115 (allele-specific RNA-Seq in WT and Dnmt3l matKO morulae, https://www.ncbi.nlm.nih.gov/geo/query/acc.cgi?acc=GSE130115),
GSE116713 (allele-specific RNA-Seq in WT and Eed matKO morulae, https://www.ncbi.nlm.nih.gov/geo/query/acc.cgi?acc=GSE116713).

Field-specific reporting

Please select the one below that is the best fit for your research. If you are not sure, read the appropriate sections before making your selection.

☒ Life sciences ☐ Behavioural & social sciences ☐ Ecological, evolutionary & environmental sciences

For a reference copy of the document with all sections, see nature.com/documents/nr-reporting-summary-flat.pdf

Life sciences study design

All studies must disclose on these points even when the disclosure is negative.

Sample size	No explicit sample-size calculation was performed, but our experimental design was guided by common community standards. For RNA-seq, we compared allelic ratios across 5 forward and 3 reverse crosses after removing two low-quality samples (see below). For WGBS, we compared two androgenote, parthenogenote, and biparental blastocysts each. The reliable detection of 23 out of 24 known GL-DMRs within a set of 859 DMRs illustrates the reliability of the WGBS dataset.
Data exclusions	RNA-seq samples 4 and 6 were removed from the analysis pipeline due to low alignment rate (less than 5 % of reads mapping to the reference genome) and low sequencing depth (too low number of total reads), respectively.
Replication	Replication attempts are reported in detail in Supplementary Dataset 3.
Randomization	No specific measures were taken regarding randomization. Randomization of sample allocation is not a standard in the field.
Blinding	Where necessary, sample collection and analyses were performed by different researchers on multiple occasions but there was no formal blinding, which is not a standard in the field.

Reporting for specific materials, systems and methods

We require information from authors about some types of materials, experimental systems and methods used in many studies. Here, indicate whether each material, system or method listed is relevant to your study. If you are not sure if a list item applies to your research, read the appropriate section before selecting a response.

Materials & experimental systems

n/a	Involved in the study	
☐	☒	Antibodies
☐	☒	Eukaryotic cell lines
☒	☐	Palaeontology and archaeology
☐	☒	Animals and other organisms
☒	☐	Human research participants
☒	☐	Clinical data
☒	☐	Dual use research of concern

Methods

n/a	Involved in the study	
☒	☐	ChIP-seq
☒	☐	Flow cytometry
☒	☐	MRI-based neuroimaging

Antibodies

Antibodies used	rabbit anti-Oct4 ChIP (Abcam ab19857), 1:100 (v/v) dilution; mouse anti-Cdx2 (BioGenex, clone CDX2-88), 1:100 (v/v) dilution.

Validation	All of the antibodies used in this work are commercially-validated and widely-used.

Eukaryotic cell lines

Policy information about cell lines

Cell line source(s)	The following ESC lines were used in this study:<br>Kind gift from Austin Smith: ES-f1 (Rex1::GFPd2 reporter cell line); ES-f2 at (129/B6 F1 hybrid female line); ES-m1 (E14TG2a male ESC line); ES-m2 (male 129 derived ESC line)<br>Derived by authors: ES-m3 (male ES cell line of mixed background carrying a floxed, intact Mek1 allele); phaES1 (pha Rex1::GFP reporter ESC line, 129 background); phaES2 (phaESC line 'P1' from a 129 background); phaES3 (phaESC line 'T8', carrying a constitutive tdTomato reporter from a 129 background); phaES4 (phaESC line 'H129-1' from a 129 background); ahaES1 (ahaESC line 'A6GFP' from a 129 background, carrying a constitutively active GFP transgene); ahaES2 p8 (ahaESC line 'A7' from a 129 background); ahaES3 p8 (ahaESC line A11 from a 129 background).
Authentication	Authentication was performed based on fluorescent markers and determination of cell cycle profiles where possible.
Mycoplasma contamination	All cell lines in the lab are regularly tested for mycoplasma contamination using commercial colorimetric tests. All cell lines used in this study were mycoplasma negative during all tests.
Commonly misidentified lines<br>(See ICLAC register)	No commonly misidentified cell lines were used in this study.

Animals and other organisms

Policy information about studies involving animals; ARRIVE guidelines recommended for reporting animal research

Laboratory animals	Mouse (Mus musculus domesticus and M. musculus castaneus), male and female, 8-12 weeks old. M.m. domesticus strains were B6D2F1 (C57BL/6 [B6] x DBA/2) or related.
Wild animals	n/a
Field-collected samples	n/a
Ethics oversight	Experiments involving animals were performed in accordance with local and national statutes including the University of Bath Animal Welfare Ethical Review Body, protecting animals in experimental research and complied with the UK Animals (Scientific Procedures) Act, 1986 and its embodiments.

Note that full information on the approval of the study protocol must also be provided in the manuscript.
